# Machine Learning and Arrhythmia: Advances in Atrial Fibrillation Detection and Management

**DOI:** 10.1007/s11883-025-01366-z

**Published:** 2025-11-28

**Authors:** Vahid Yazdi, Vishnu Kadiyala, Sumeet S. Chugh

**Affiliations:** 1 Artificial Intelligence in Medicine Research Center and Smidt Heart Institute, Cedars-Sinai Health System, Los Angeles, CA USA; 2 Smidt Heart Institute, Cedars-Sinai Health System Bard Pavilion, Suite A3100 127 S. San Vicente Blvd, Los Angeles, CA 90048 USA

**Keywords:** Machine learning, Atrial fibrillation, Artificial intelligence, Electrophysiology, Prediction, Prevention, Risk

## Abstract

**Purpose of Review:**

In this paper we review recent advancements in the diagnosis and management of atrial fibrillation through machine learning (ML).

**Recent Findings:**

Machine learning models developed from clinical records, electrocardiograms (ECGs) as well as data from implantable and wearable devices can now detect and even predict new-onset atrial fibrillation. Other models have improved prediction of stroke risk, increased the success of electrical cardioversions and facilitated catheter ablation of AF.

**Summary:**

Machine learning presents exciting new opportunities to enhance detection and management of atrial fibrillation. However, these developments need to be weighed against considerations of generalizability, equity, and transparency of these models for real-world utilization in clinical practice. We suggest targeted approaches for evaluation and utilization of ML models to allow for informed clinical implementation.

## Introduction: Knowledge Gaps in AF Detection and Management

Atrial fibrillation (AF) is the most common sustained cardiac arrhythmia and is associated with a spectrum of comorbidities including advanced age, heart failure, valvular disease, hypertension, obesity, obstructive sleep apnea and diabetes mellitus [1–3]. AF contributes significantly to stroke and heart failure and is also linked to dementia, sudden cardiac death and increased overall mortality [4, 5]. In the US, lifetime risk of AF is 1 in 3 for white individuals and 1 in 5 for black individuals, with the current prevalence projected to more than double by 2050 [6, 7]. As our population ages, AF continues to loom as an increasing public health burden [1, 8]. AF management will place a progressively greater financial strain on our healthcare system, since the mean total cost per patient per year with incident AF is $27,896 more than those without AF [9].

A more recent study has suggested that early management of AF can reduce incidence of stroke, heart failure and recurrent AF, making a timely diagnosis crucially important [10, 11]. However, the clinical presentation of AF can be heterogenous. While often associated with common symptoms such as dyspnea, chest pain, dizziness, fatigue, palpitations, a large proportion of patients with AF are asymptomatic [12]. Therefore, there is a need for predictive screening tools to assist with expedient diagnosis and subsequent management of AF. Artificial intelligence (AI) tools, specifically machine learning (ML), has shown significant promise to address this need.

## What is Machine Learning?

Machine learning (ML) is a type of artificial intelligence that performs automated learning from data sets with the end goal of defining relationships between input and output data [13]. Classically, in computer science, a program is instilled with rules and pre-defined relationships which guide exactly how to process data and make decisions. ML, in contrast, mimics human intelligence by self-defining the relationships between inputs (features) and their corresponding outputs (labels). It does this after a learning period in which features and labels are used to train the model, and machine learning models can make associations between variables without being explicitly programmed to do so. In this way, it can make accurate predictions based on new, unseen data. This type of machine learning is known as supervised ML [13]. Alternatively, unsupervised ML uses unlabeled data to discover patterns and structures within the data.

Deep learning (DL) is a potent class of models used within the framework of supervised ML that utilizes hidden layers –intermediate layers between inputs and outputs that allow for weighing multiple variables – to create a deep neural network (DNN) [14]. DNNs are artificial neural networks that can learn complex, hierarchical relationships between features and labels. These networks are well-suited to learn from complex, heterogenous data sets which they can then use to adjust internal parameters (weights) through a process called backpropagation. Backpropagation uses total error obtained from current output which is passed backwards from the labels to the features and updates the weight of each variable to reduce error [15]. This is a unique process that allows less manual engineering in the initial DNN model and increases self-optimization by minimizing loss. However, this complex underlying architecture requires larger initial data sets and often lacks explainability, which is especially important in healthcare.

Explainable AI (XAI) is an ongoing attempt to enhance the explainability of DL systems. Saliency mapping is a type of XAI which is used to improve the explainability of convolutional neural networks (CNNs – a type of DNN) [16]. It identifies the most important datapoints in the input that push the model to reach its output [17]. AF is a complex disease process that involves multiple underlying substrates (i.e. hypertension, structural heart disease) and acute triggers (i.e. rapidly firing ectopic foci, increased automaticity) [18]. Current ML models are aimed at predicting AF diagnosis through clinical characteristics, electrocardiograms (ECGs), wearable devices and helping with risk stratification and management. In the future, DNNs may ostensibly define new relationships within the complex pathophysiology of AF.


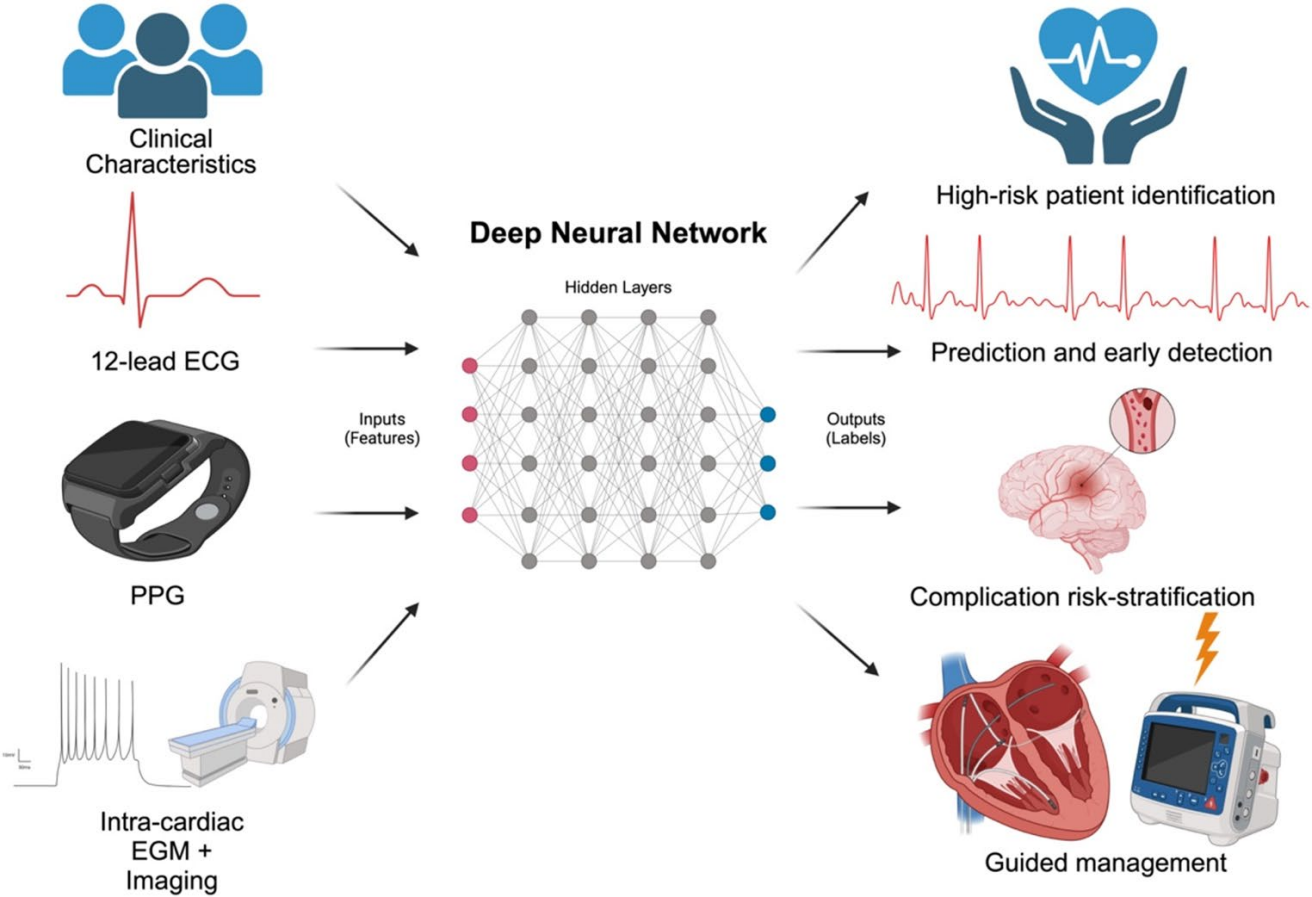


**Central Illustration.** Machine learning, specifically deep learning, has demonstrated great promise in detection, risk-stratification, and management of atrial fibrillation. These models are based on clinical characteristics, electrocardiograms, photoplethysmography, intra-cardiac electrograms and imaging. ECG represents electrocardiogram; PPG, photoplethysmography; EGM, electrogram. Created in BioRender. https://BioRender.com/4vowvqq.

## AF and Machine Learning: Current Knowledge

### Diagnosis

Several predictive scores for new-onset AF have been proposed in the past two decades, that are based on clinical characteristics [19, 20]. CHARGE-AF is a 5-year score for incident AF prediction using age, race, height, weight, systolic and diastolic blood pressure, current smoking, use of antihypertensive medication, diabetes mellitus, and history of myocardial infarction and heart failure. The score has shown adequate discrimination with additional validation. Despite its promise, it is not widely used clinically as it was primarily developed and validated using elderly, European cohorts, can be difficult to calculate, and lacks widespread acceptance [21, 22]. This is a common problem among many traditional statistically derived scores.

**Fig. 1:**
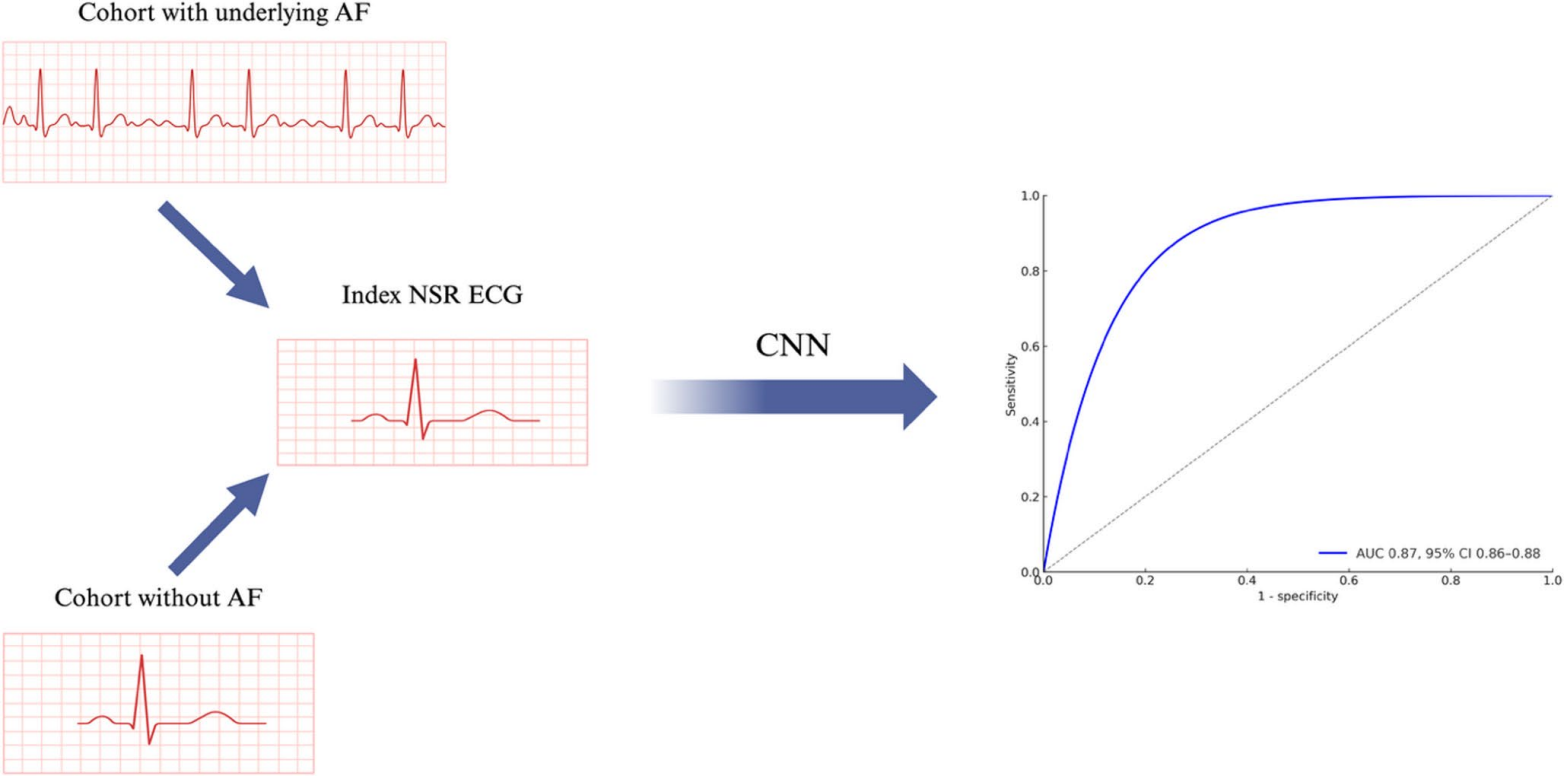
Development of a CNN to detect underlying paroxysmal atrial fibrillation based on a single 12-lead normal sinus rhythm ECG with an area under the curve of 0.87. AF indicates atrial fibrillation; AUC, area under curve; NSR, normal sinus rhythm; CNN, convolutional neural network;ECG electrocardiogram. Created in BioRender. https://BioRender.com/9i02h2g

More recent studies have developed AF prediction ML models from sinus rhythm ECGs based on 10-second, 500 Hz, and12-lead ECG samples. In a landmark study, Attia et al., developed a deep learning model trained on 649,931 ECGs in normal sinus rhythm to predict underlying paroxysmal AF. Individual normal sinus ECGs were used as input with a resulting area under the receiver operating curve (AUROC) of 0.87 (Fig. 1). Including an input of all ECGs acquired during the first month of each patient’s window of interest increased the AUROC to 0.90 [23].

**Fig. 2:**
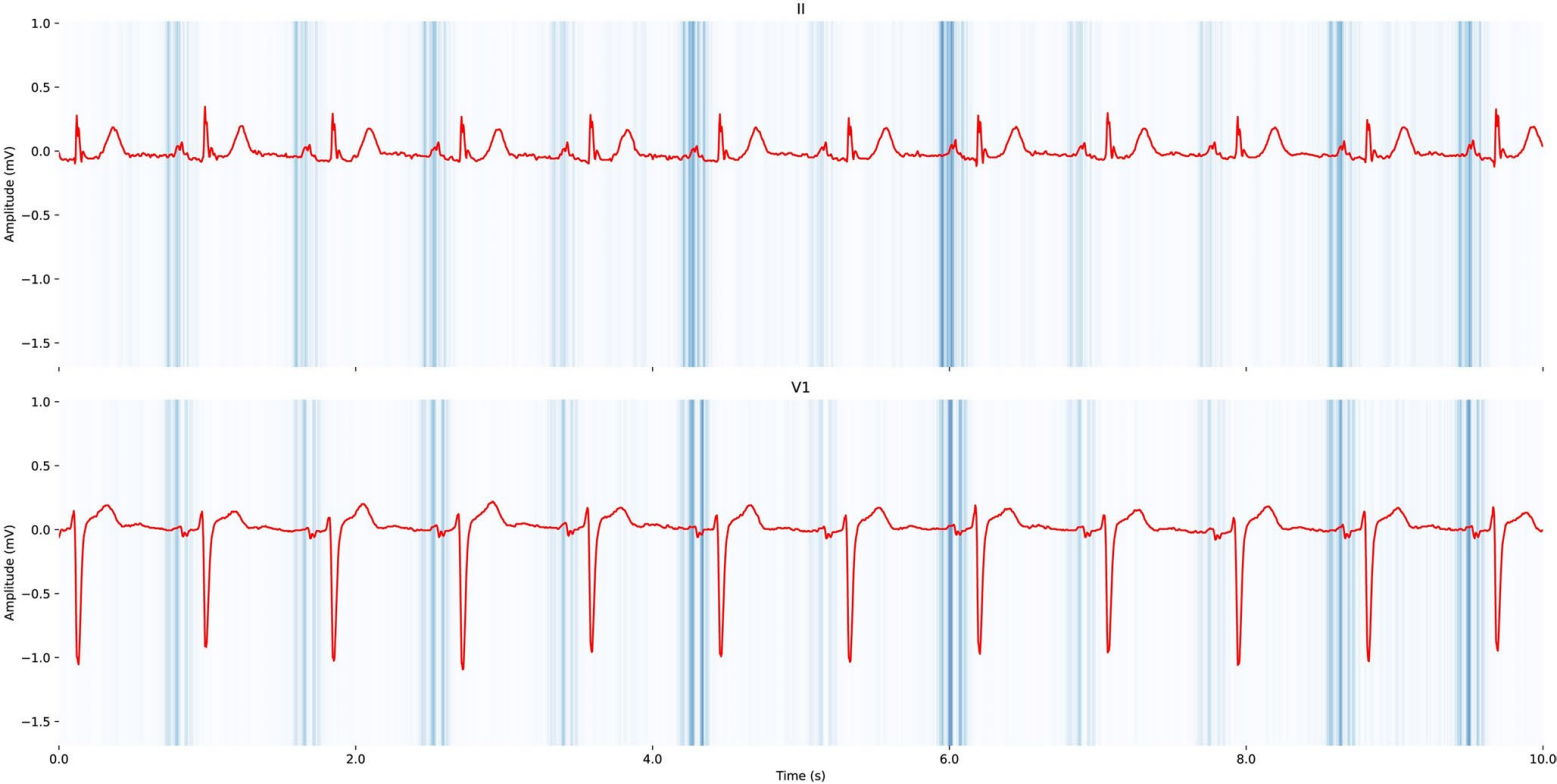
Saliency mapping of ECG tracings (leads II and V1) which visualizes the weight of segments for prediction of incident atrial fibrillation using electrogram-based deep learning. The color gradient from light (low saliency) to dark (high saliency) represents the weight of each segment in the model’s output prediction. Notably, there is high saliency around the P-wave indicating that is the importance of this ECG feature for predicting atrial fibrillation. Reprinted from Jabbour et al. [26] with permission

Subsequently, a DNN designed by Raghunath et al., to predict incident AF within 1 year of a normal sinus ECG demonstrated good discrimination (AUROC, 0.85) with a number needed to screen of 9 to find 1 new case of AF. This study included a subset analysis which showed superior performance (AUROC, 0.84) when compared to CHARGE-AF score (AUROC, 0.79) for patients in the cohort with adequate data for CHARGE-AF calculation. Raghunath et al. also reported that 62% of patients who had an AF-related stroke within 3 years of the input ECG were predicted to be at high-risk for new-onset AF based on the DNN, suggesting a possible future prognostic function of ML in AF management [24]. Khurshid et al., subsequently published a convolutional neural network (CNN) integrating ECG-AI and CHARGE-AF together to create CH-AI on 50,461 patients receiving longitudinal primary care at Massachusetts General Hospital, plus external validation in two additional cohorts. CH-AI had an AUROC of 0.838 – outperforming its constituent parts ECG-AI (AUROC, 0.823) and CHARGE-AF (AUROC, 0.802). This suggests a complementary element between clinical characteristics and ECG-AI models which can improve discrimination. Saliency mapping of this CNN revealed the output prediction was most influenced by the ECG-P wave and surrounding regions [25]. This finding was also reported by Jabour et al., with improvement in AUROC for predicting incident AF when ECG-AI was combined with CHARGE-AF and polygenic clinical scores [26]. Saliency mapping was again, mostly influenced by the ECG-P wave and surrounding regions (Fig. 2).

This finding is likely reflective of atrial cardiopathy – extracellular matrix and electrical remodeling in the left atrium (LA) which may develop well before incident atrial fibrillation as demonstrated in PREDICT-AF [27–29]. Choi et al., trained two CNN ML-models from patients at Samsung Medical Center in Korea – one for single ECG input (as previous studies had done) and one for two serial ECG input [30]. The serial ECG model for two normal sinus ECGs within 1 month of each other (AUROC, 0.947) significantly outperformed the single ECG model (AUROC, 0.910) for predicting new-onset AF. Notably, the serial ECG model AUROC improved to 0.964 when the interval between ECGs extended to 14 to 23 months. Choi et al., then performed Shapley Additive Explanations (SHAP) analysis for XAI and found that elements related P-wave morphology were among the top 5 ECG parameters and age and sex were more important than any individual ECG parameter for predicting outcomes [30]. This further supports the hypothesis that subtle ECG changes resulting from LA remodeling become more pronounced over time and can result in more accurate ML predictions of new-onset AF, a hypothesis that correlates with clinical understanding of the pathogenesis of AF [31]. It also solidifies the importance of utilizing ECG-AI systems alongside clinical characteristics (i.e. age, sex, comorbidities) to develop the most accurate AF ML-prediction systems.

AF can also be diagnosed from indwelling pacemakers or implantable cardioverter defibrillators (ICD) based on electrograms (EGM) recorded by these implanted electronic devices. However, automated device detection of AF can be suboptimal. These devices typically detect AF, atrial flutter or tachycardia (AT) by changes in rate or regularity, which can result in misclassification of organized tachycardias or even premature atrial ectopic beats as AF. Rodrigo et al., addressed this problem by devising a DL system that can accurately distinguish AF from organized ATs even in the presence of overlap in rate or regularity measurements. This DL algorithm identified AF with AUROC of 0.97 for unipolar EGMs and 0.92 for bipolar EGMs showing a promising use of ML in implantable devices [32].

DL models utilizing transthoracic echocardiograms (TTEs), which contain structural information, have also been utilized to identify occult AF. Yuan et al., created a two-stage CNN which distinguished whether TTEs were performed in sinus rhythm or AF and then predicted which of the sinus rhythm TTEs had experienced AF within the past 90 days [33]. Discrimination of TTEs in AF from those in sinus rhythm was achieved with an AUROC of 0.96 and among TTEs in sinus rhythm, and the model predicted concomitant paroxysmal AF with an AUROC of 0.74. Then a combined model utilizing TTE and ECG DL models outperformed the ECG model alone (AUROC 0.81 vs. 0.79) suggesting TTE has a potential role in improving detection of patients with active or occult AF [33].

Another area of ML-based screening for AF which has garnered significant attention is wearable devices such as smartwatches, mainly using photoplethysmography (PPG) [34]. PPG measures volumetric changes in blood flow based on the intensity of reflected light measured on the skin surface [35]. Algorithms that use this pulse wave data, largely based on R-R interval times, to detect and even predict transition to AF have been developed [35–37]. The first study that utilized this methodology was the Apple Heart Study, which recruited 419,297 participants with an Apple Watch and without self-reported history of AF. Among the 2,161 patients who received an abnormal pulse notification, 34% were shown to have AF on subsequent gold-standard ECG patch readings within 7 days. However, it should be noted that only 20.8% of patients who were notified of an irregular pulse returned an ECG patch. Among the subgroup of patients who both received notifications from the Apple Watch and returned the ECG patch, the positive predictive value for detection of AF was 84% [38]. The Fitbit Heart Study had a similar design – 455,699 participants without AF enrolled and 1,057 received an irregular pulse notification and were mailed an ECG patch to be worn for a week. Among the 1,057 participants, 32.2% were found to have AF with a PPV of 98.2% [39]. Similarly, MAFA-II enrolled >180,000 individuals who used smart devices and found that among those with suspected AF based on PPG, 87% were confirmed with a PPV of 95.1% [40]. Of note, the specific algorithms used in these studies are proprietary and thus, remain undefined. However, these are likely related to the R-R interval, which is what previously developed PPG-algorithms utilized to predict AF [35–37]. Based on these reports, ML models show promise for prediction and screening of AF. Some of these studies also demonstrate potential for advancement of AF pathophysiology.

### Prognostic (Risk Stratification) and Management Models

#### Stroke

The most feared complication of AF is thromboembolism and stroke [4, 5]. The CHA2DS2-VASc score and HAS-BLED are well-established clinical risk scores which guide anticoagulation use in AF [41, 42]. ML models are currently being developed to further risk stratify patients beyond these clinical risk scores which have low sensitivity and modest positive predictive value in stroke prediction [43, 44].

Jung et al., used data from 754,949 patients with AF from the Korean National Health Insurance database to identify 48 features significantly associated with ischemic stroke through regression analysis. They utilized these features to create a DNN from 150,989 patients and confirmed prediction of ischemic stroke with an AUROC 0.727 – compared to CHA2DS2-VASc AUROC of 0.651 [45]. Joddrell et al., utilized multiple ML models on patients with non-valvular AF and improved 3-year prediction of stroke with linear discriminant analysis (AUROC 0.654), as compared to CHA2DS2-VASc (AUROC 0.535) [46, 47]. A meta-analysis of 13 studies with a total of 7,094,555 participants revealed a mean AUROC of 0.73 for ML models, indicating an overall average predictive performance [48]. Therefore, it is likely that ML models for risk stratification can be utilized alongside clinical risk scores to further improve clinical decisions regarding anticoagulation. However, since the level of performance is moderate, there remains room for improvement.

#### Cardioversion

Identifying patients who will benefit from cardioversion is challenging in clinical practice as there is a high rate of recurrence [49]. Vinter et al., developed a sex-specific AI-model aimed at predicting success of electrical cardioversion in AF. Discrimination was similar for machine learning (AUROC 0.59 for women, 0.56 for men) and LR models (0.60 for women and 0.59 for men) [50]. Subsequently, Kwon et al., improved upon this performance by using 12-lead ECG data combined with other clinical features to develop an XGBoost-based model (ML model that uses gradient boosted decision trees) with an AUROC of 0.63 [51]. While only moderately predictive, these studies represent important initial steps towards utilizing ML to predict AF recurrence after cardioversion.

#### Ablation

Catheter ablation therapy for AF is becoming increasingly utilized as initial AF management - it is now a Class 1 indication in selected patients to reduce arrhythmia recurrence and burden [52–54]. Recent ML models have been developed to assist with predicting post-ablation success rates and planning these complex procedures.

Budzianowski et al., created a ML model for predicting arrhythmic recurrence within the first year after pulmonary vein (PV) isolation catheter ablation. The model utilized 12 clinical variables to achieve an AUROC of 0.75 for prediction of one-year AF recurrence. SHAP analysis demonstrated early recurrent AF (within 3 months of ablation) was the most important factor in the model. Other highly weighted variables included TSH, HAS-BLED score, statin therapy, and fibrinogen [55]. Tang et al., then developed a CNN for predicting 1-year AF recurrence after ablation utilizing a composite model of LA intracardiac EGM, ECG signals and clinical features with an AUROC of 0.859 – outperforming EGM-alone and ECG-alone CNNs (AUROC 0.731 and 0.767 respectively) [56]. Pulmonary vein (PV) remodeling has also been associated with AF recurrence [57, 58]. A recent study developed a DL model to evaluate primary and secondary PV branch segmentation. Recurrent AF cases exhibited greater surface complexity in the primary PV area with a classification for AUROC of 0.73 – signifying DL also has a role in better understanding the pathophysiology of AF [59].

**Fig. 3:**
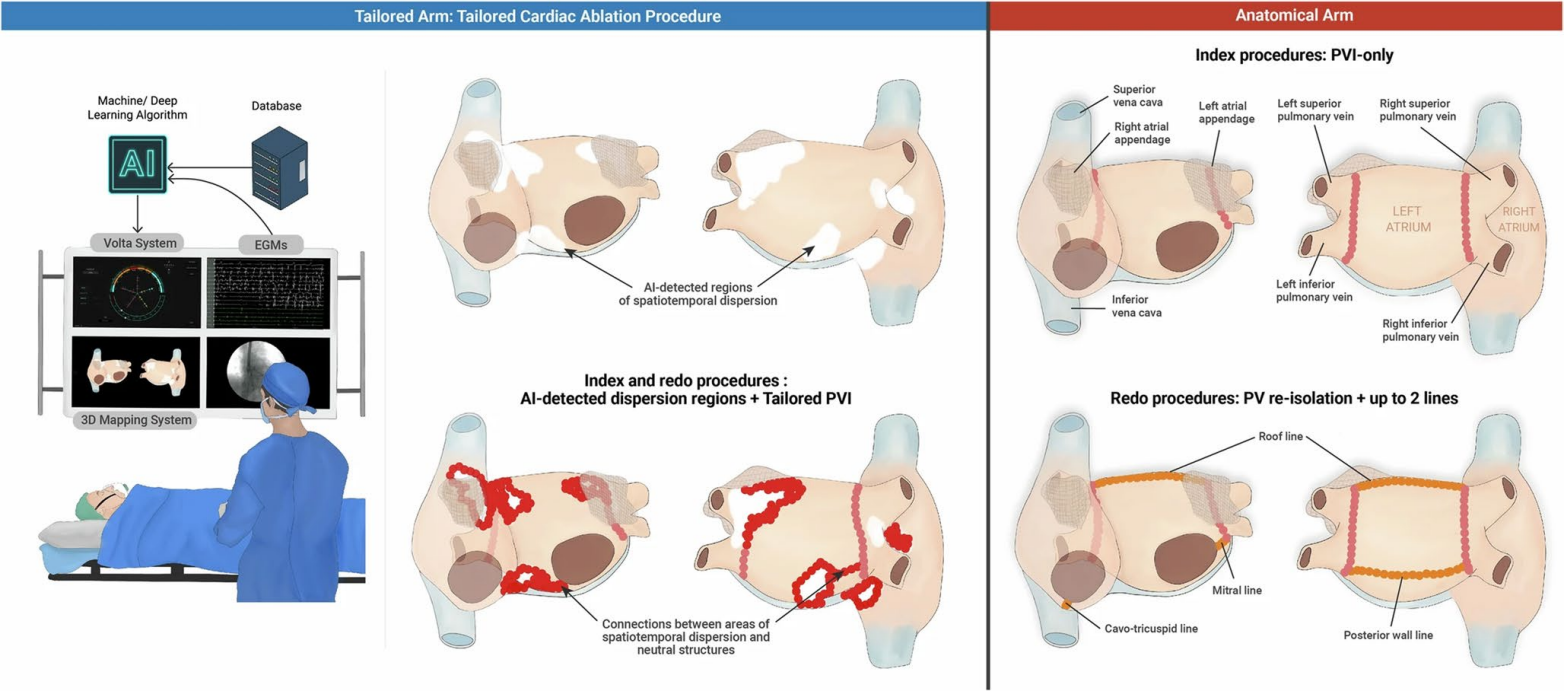
Both randomization arms underwent mapping of the left and right atria with the Volta system. The AI classifier then identified spatio-temporal dispersion using intra-cardiac electrograms. The tailored arm ablated dispersion areas (white regions) until AF terminated to sinus or until all dispersed areas were eliminated or isolated (reg tags). Then pulmonary vein isolation was completed (pink tags). The anatomical arm isolated antral pulmonary veins (pink tags) until bidirectional block was achieved. AI indicates artificial intelligence; PVI, pulmonary vein isolation. Reprinted from Deisenhofer et al. [63] without any changes under Creative Commons Attribution 4.0 International License (http://creativecommons.org/licenses/by/4.0/)

In addition to PV remodeling, non-pulmonary vein (NPV) triggers are an independent predictor of AF recurrence and are responsible for nearly half of the arrhythmias recurring in patients requiring repeat ablations [60]. Pulmonary vein computed tomography (PVCT) slices from 358 patients with nonrecurrent paroxysmal AF were used in a DNN to create a prediction model to identify NPV triggers. The AUROC for each patient receiving a PVCT image was 0.88, and could provide electrophysiologists with additional information to assist with pre-ablation planning [61]. Focal sources are also potential targets for AF catheter ablation and can be time-consuming and difficult to identify when unipolar EGMs are numerous and complex. A CNN was trained on raw unipolar EGMs to classify focal source and trigger sites with an AUROC of 0.923 [62]. This AUROC displays similar performance to focal source and trigger re-classification by Cardiologists – suggesting that DL could have a role in improving the efficiency of real-time focal source detection for targeted ablations. Notably, TAILOR-AF, a recent RCT randomized patients with drug-refractory persistent AF to two groups: a tailored ablation using targeted areas of spatiotemporal dispersion detected from intra-cardiac EGMs by an AI classifier in addition to PV isolation, versus a conventional anatomical PV isolation group. The group with a tailored cardiac ablation procedure had a higher success rate of AF termination and AF freedom at 12 months (88% of patients) versus the PV isolation group alone (70% of patients) [63]. 50/180 and 38/177 in the tailored and conventional arms, respectively underwent repeat procedures (no statistically significant difference). However, 86% of the tailored arm repeat procedures were for atrial tachycardias and 76% of the conventional arm repeat procedures were for AF (Fig. 3).

The growing body of literature on ML models for catheter ablation planning as well as outcome prediction remains a promising direction for AF management.

## Challenges for Development and Deployment of ML Algorithms for AF

While ML has shown promise in diagnosis and management of AF, there are significant limitations preventing its full integration into clinical practice. ML itself has inherent obstacles that impede broad utilization by clinicians. Firstly, the black box or grey box model in which many AI systems operate offers little to no explanation behind algorithm outputs [64]. Saliency mapping and other XAI represent attempts to remedy this limitation by providing some insight into the weight of hidden layers. However, these methods do not offer complete explanations. Explainability is an issue that invokes legal, ethical and societal questions; and therefore, poses a high barrier for acceptance before ML can be widely implemented without improvements in the opacity of these DL algorithms [65].

Additionally, DL systems require a large, representative, diverse data set to train and minimize the difference between the trained and applied population. While many of the studies we reviewed have excellent discrimination at the institutions they were developed, they have not been externally validated at different institutions with different patient populations. For example, in the landmark study by Attia et al., the patient selection for AF prediction was hospitalized patients with cardiac comorbidities and a high volume of sinus ECGs, which may not necessarily be generalizable [23, 66].

Non-standardized electronic health record (EHR) systems also invoke the data maxim, “garbage in, garbage out,” [13]. Therefore, each institution must have its own standardized data on its own unique population, but certain institutions may not have the necessary volume of data to create accurate ML models. This leads to the question of potentially sharing large-data sets between institutions for model training which then raises more concerns about data sharing and privacy. Clearly, the data sets for these ML models are, and will continue to be a significant limitation to their widespread clinical application, especially when compared to non-medical ML models, which can use data sets magnitudes greater in size for increased model fidelity.

Another challenge relates to universal access of ML technology, especially PPG gathered from wearable devices. Most of the studies that reported findings from wearable devices were skewed toward individuals in higher-income categories, who were able to afford wearable devices and who were more concerned with their cardiovascular health [67]. Data about the utility of these devices in other socioeconomic groups is limited. This suggests possible racial and economic barriers to ML model application and adoption for detection of AF, which limits the generalizability and applicability of PPG gathered from wearable devices.

## Future Directions

ML clearly has a large role to play in both the diagnosis and management of atrial fibrillation. However, there is still some distance to go in both the construction of these ML models and their integration into clinical practice.

As of now many DL systems such as CNNs have limited explainability since the most heavily weighted features selected by the models cannot be known. As our understanding of ML continues to improve, it is possible that AI systems with increased level of explainability will emerge. Jo et al., have already designed a XAI DL model with a strong AUROC for detection of AF in a retrospective cohort (0.997–0.999) which provides a description behind the reason for the algorithm’s decision [68]. Further advancement on this front will inform clinical decision-making but may also present new avenues for improved understanding of AF at a pathophysiological level. For example, the P wave morphology detection in ECGs is a proof of our understanding of subtle atrial remodeling that predates AF. Unsupervised ML may be able to uncover other unknown variables and patterns behind the disease.

Additionally, many of the models we have reviewed are focused on single-variable features. However, when multiple variables are included, there is increased ML model accuracy. Designing models in the future that focus on multi-variable features including, but not limited to ECG, clinical characteristics, imaging and electrophysiological data may lead to more robust ML models for detection and management of AF.

ML models may be able to improve overall clinical practice for AF but these must be appropriately integrated into clinical practice. One of the main limitations of existing ML models is the lack of generalizability. Finding populations with appropriate pre-test probability to test with ML models will be crucial. The BEAGLE trial recruited patients with stroke risk factors but no known AF, who had ECGs that were stratified by AI into high-and-low risk groups. The high-risk group experienced significantly increased new AF diagnosed with a wearable device– suggesting that ML-based screening may be appropriate for patients with AF comorbidities in an outpatient setting [69]. The application of these ML models in parallel with their optimization will be an important direction for ML in AF.

## Conclusion

ML has provided a promising new avenue through which we can enhance diagnosis and optimize management of AF. ML models incorporating clinical characteristics, PPGs, ECGs, imaging, and electrophysiological data have been designed with adequate discrimation for diagnosis, prognostication, and management options for AF. However, the application of these models still faces challenges in generalizability, equity, transparency, and privacy. Future research will focus on overcoming these challenges with continued technological innovation, and will provide us with a better understanding of the real-word impact of ML in AF.

## Key References

- Rajkomar A, Dean J, Kohane I. Machine Learning in Medicine*.* N Engl J Med. 2019;380(14):1347-1358.
  - ○ This is an overview of the potential uses and limitations of machine learning in medicine.
- Attia ZI, Noseworthy PA, Lopez-Jimenez F, et al. An artificial intelligence-enabled ECG algorithm for the identification of patients with atrial fibrillation during sinus rhythm: a retrospective analysis of outcome prediction. Lancet. 2019;394(10201):861–867.
  - ○ The first AI-ECG model which was designed to detect underlying atrial fibrillation from an ECG in normal sinus rhythm.
- Khurshid S, Friedman S, Reeder C, et al. ECG-Based Deep Learning and Clinical Risk Factors to Predict Atrial Fibrillation. Circulation. 2022;145(2):122–133.
  - ○ These investigators created a CNN combining ECG-AI and CHARGE-AF to create CH-AI which outperformed its constituent parts. It was also externally validated on two additional cohorts.
- Lubitz SA, Faranesh AZ, Selvaggi C, et al. Detection of Atrial Fibrillation in a Large Population Using Wearable Devices: The Fitbit Heart Study. Circulation. 2022;146(19):1415-1424.
  - ○ The Fitbit heart study which reported a PPV of 98.2% through PPG on wearable devices.
- Jung S, Song MK, Lee E, et al. Predicting Ischemic Stroke in Patients with Atrial Fibrillation Using Machine Learning. Front Biosci (Landmark Ed). 2022;27(3):80.
  - ○ Developed an ML model which predicted ischemic stroke in a population with an AUROC better than that of CHA2DS2-VASc.
- Deisenhofer, I., Albenque, JP., Busch, S. et al*.* Artificial intelligence for individualized treatment of persistent atrial fibrillation: a randomized controlled trial. Nat Med. 2025;31(4):1286-1293.
  - ○ A recent RCT which established improved AF ablation outcomes in patients who received tailored ablation as determined by AI in addition to standard pulmonary vein isolation.

## Data Availability

No datasets were generated or analysed during the current study.
